# Increased expression levels of PIEZO1 in visceral adipose tissue in obesity and type 2 diabetes are triggered by mechanical forces and are associated with inflammation

**DOI:** 10.1186/s10020-024-01008-1

**Published:** 2024-12-20

**Authors:** Victoria Catalán, Javier Gómez-Ambrosi, Beatriz Ramírez, Xabier Unamuno, Sara Becerril, Amaia Rodríguez, Jorge Baixauli, Gabriel Reina, Ana Sancho, Camilo Silva, Javier A. Cienfuegos, Gema Frühbeck

**Affiliations:** 1 Metabolic Research Laboratory, Clínica Universidad de Navarra, Pamplona, Spain; 2 CIBER Fisiopatología de la Obesidad y Nutrición (CIBEROBN), Instituto de Salud Carlos III, Pamplona, Spain; 3 Obesity and Adipobiology Group, Instituto de Investigación Sanitaria de Navarra (IdiSNA), Pamplona, Spain; 4 Department of Surgery, Clínica Universidad de Navarra, Pamplona, Spain; 5 Department of Microbiology, Clínica Universidad de Navarra, Pamplona, Spain; 6 Biomedical Engineering and Science Department, University of Navarra, TECNUN School of Engineering, San Sebastián, Spain; 7 Department of Endocrinology and Nutrition, Clínica Universidad de Navarra, Pamplona, Spain

**Keywords:** PIEZO1, VAT, Mechanotransduction, Inflammation, Obesity, Type 2 diabetes

## Abstract

**Background:**

PIEZO1 has emerged as a mechanoreceptor linked with adipogenesis, adipose tissue (AT) inflammation and insulin resistance. We aimed to determine the impact of obesity and obesity-associated type 2 diabetes (T2D) as well as mechanical compression forces on the expression of *PIEZO1* in visceral AT (VAT) and its relation with inflammation.

**Methods:**

Blood and VAT samples were obtained from 100 volunteers. Static compression studies in VAT explants were performed to study the PIEZO1 response. The effect of bariatric surgery on the expression of *Piezo1* was assessed in a rat model of diet-induced obesity.

**Results:**

Obesity and obesity-associated T2D increased (*P* < 0.01) gene expression levels of *PIEZO1* in VAT mainly due to adipocytes. *SWELL1* and key markers of inflammation (*NLRP3*, *NLRP6*, *IL1B*, *IL18* and *IL8*) were also upregulated in VAT in obesity and T2D being significantly associated (*P* < 0.01) with *PIEZO1* levels. We further showed that the static compression of VAT explants promoted an upregulation of *PIEZO1* (*P* < 0.01) and *SWELL1* (*P* < 0.01) expression levels together with a strong increase in the expression and release of key inflammatory mediators. The treatment of THP-1-derived macrophages with the secretome of adipocytes from patients with obesity upregulated (*P* < 0.001) *PIEZO1* levels. Rats undergoing bariatric surgery exhibited decreased (*P* < 0.01) expression levels of *Piezo1* in the epididymal AT.

**Conclusions:**

Static compression triggered an upregulation of *PIEZO1* in VAT explants together with a strong inflammation. In addition, the increased expression of *PIEZO1* in VAT in obesity and obesity-associated T2D, primarily attributable to adipocytes, is closely associated with *SWELL1* and inflammatory markers.

**Supplementary Information:**

The online version contains supplementary material available at 10.1186/s10020-024-01008-1.

## Background

The extracellular matrix (ECM) of the adipose tissue (AT) constitutes a complex network functioning not only as a mechanical support but also as a structure responsible for the regulation of mechanotransduction and adhesion signaling pathways (Sun et al. 2023). Since obesity is characterized by AT expansion, the remodeling and reorganization of the ECM is required for adipocyte hypertrophy and hyperplasia together with immune cell infiltration, inflammation and fibrosis (Gliniak et al. 2023), promoting important modifications in its biomechanical and tensile properties (Alkhouli et al. 2013; Unamuno et al. 2022; Iskratsch et al. 2014) and, therefore, changes in mechanotransduction responses (Janmey et al. 2020). In this context, adipocytes sense changes in mechanical forces within the AT microenvironment including stretching and compression as well as internal pressure due to the store of triglycerides and respond by transducing these stimuli into biochemical signals through the activation of mechanosensitive ion channels (Humphrey et al. 2014; Romani et al. 2021).

Mechanosensitive channels are integral membrane proteins expressed in multiple tissues that by switching between the closed and open conformations, allow ions and other solutes to flow across the cellular membranes controlling important physiological functions (Martinac 2004; Ridone et al. 2019). Coste et al. (2010) identified in 2010 the Piezo family, a new class of mammalian mechanically activated cation channels comprising two members, PIEZO1 and PIEZO2, with a ∼ 50% identity at the aminoacid level. PIEZO1 is permeable to monovalent and most divalent ions (Gnanasambandam et al. 2015) and, therefore, its opening induces an inward-depolarizing current (Coste et al. 2010). PIEZO1 can be found in the plasma membrane, being strongly dependent on the “lipid rafts” and influenced by membrane tension as well as in the nucleus, displaying other functions beyond mechanotransduction (Ridone et al. 2019; Gudipaty et al. 2017; Haselwandter and MacKinnon 2018). PIEZO1 also operates in conjunction with other depolarizing membrane channels, including SWELL1 (a volume-regulated anion channel, VRAC), the calcium-activated potassium cannel KCa3.1 or members of the transient receptor potential channels family (Martinac 2004; Martinac and Poole 2018; Ran et al. 2023). Caveolae, small invaginations of the plasma membrane, regulate mechanotransduction, signal transduction, and lipid metabolism (Frühbeck et al. 2007). Recent studies suggest that caveolin-1 (CAV1), a crucial scaffolding protein involved in the formation of caveolae, regulate the activity of PIEZO1 by influencing its response to mechanical stimuli or its localization in the membrane (Diem et al. 2020). While PIEZO2 is mainly expressed in a subset of sensory neurons, PIEZO1 is expressed in non-sensory neurons and other tissues (Murthy et al. 2017; Zong et al. 2023). In this sense, PIEZO2 is implicated in mediating sensory mechanotransduction and PIEZO1 is related to broad roles in multiple physiological processes including epithelial homeostasis, cardiovascular and brain mechanotransduction, bone formation, regulation of red blood cell volume as well as cell migration and differentiation (Murthy et al. 2017; Wang et al. 2022). In addition to these functions, Atcha et al. (2021) elegantly demonstrated the relationship between PIEZO1, substrate stiffness and inflammation: They demonstrated that the activation of PIEZO1 by stiffness or by interferon-γ and lipopolysaccharide (LPS) enhanced Ca^2+^ influx together with actin polymerization, activating the transcription factor NFκB and promoting the upregulation of key inflammatory markers (Atcha et al. 2021). Other inflammation-related mechanisms have been proposed, with PIEZO1 functioning as a co-receptor of TLR4 to promote cytoskeleton remodeling, Ca^2+^ influx and the activation of CaMKII-Mst1/2-Rac axis (Geng et al. 2021). Interestingly, PIEZO1 also enabled myeloid cells to sense their physical environment promoting a shift toward a proinflammatory profile through mechanical stimulation alone, independently of classical pattern recognition receptors (PRR) signaling (Solis et al. 2019). Remarkably, the NLRP3 inflammasome, a master regulator of inflammatory pathways, can be activated by different endogenous DAMPs, including Ca^2+^ among others (Gong et al. 2018). In this line, the activation of the NLRP3 inflammasome by PIEZO1 in nucleus pulposus cells (Sun et al. 2020) as well as in macrophages (Ran et al. 2023; Fish and Kulkarni 2024) and intestinal epithelial cells (Liu et al. 2023) has been described.

AT-associated chronic inflammation and fibrosis in obesity are responsible for the development of many comorbidities, including type 2 diabetes (T2D) (Gómez-Ambrosi et al. 2006; Unamuno et al. 2018). In mammals, Piezo1 has been recognized as an important regulator for glucose-stimulated insulin secretion in β cells (Ye et al. 2022). In addition, Piezo1-mediated stomach stretch and satiety have been described in animal models (Min et al. 2021) and recently, the role of Piezo1 in appetite control by the mechanical regulation of ghrelin production in X/A-like cells has also been proposed (Zhao et al. 2024).

Since emerging evidence links PIEZO1-mediated mechanotransduction and signaling pathways to AT biology, inflammation and metabolic regulation, we hypothesize that PIEZO1 is dysregulated in visceral AT (VAT) in the context of obesity and obesity-associated T2D, being activated by mechanical forces and influencing the development of AT inflammation. Therefore, we aimed (i) to characterize the transcript levels of *PIEZO1* in VAT in obesity and obesity-associated T2D together with its relationship with *NLRP3*, *SWELL1* and caveolin 1 (*CAV1*), (ii) to explore the role of compression forces on the expression of *PIEZO1* and key inflammation- and ECM-related factors, (iii) to analyze the impact of weight loss on the expression of *Piezo1* in an animal model of diet-induced obesity (DIO) and (iv) to evaluate the effect of the adipocyte secretome from patients with obesity in the expression of *PIEZO1* in THP-1-derived macrophages.

## Material and methods

### Study population

The study included 100 samples obtained from 59 females and 41 males, consisting of normoglycemic patients with normal weight (NW) (n = 20) and people diagnosed with obesity (OB) (n = 80). Body mass index [(BMI), calculated as weight in kg divided by the square of height in m] and body fat [(BF), determined by using air displacement plethysmography (BodPod^®^, Life Measurements, COSMED, Italy)] were used to define obesity. People diagnosed with obesity (OB) were divided into three subgroups: those with normoglycemia (NG, n = 25) and those with impaired glucose tolerance (IGT, n = 31) and newly diagnosed type 2 diabetes (T2D, n = 24). Patients with IGT and T2D were grouped together (OB-IGT + T2D, n = 55). The study adhered to the guidelines set by the American Diabetes Association to define IGT and T2D (Practice et al. 2024). It is important to note that people with T2D were not receiving any pharmacotherapy that could affect their natural insulin levels. VAT biopsies were collected from people with obesity during laparoscopic Roux-en-Y gastric bypass (RYGB) surgery and from volunteers with NW during laparoscopic Nissen funduplication. The research protocol followed the guidelines outlined in the Declaration of Helsinki and was approved by the Ethical Committee of the Universidad de Navarra (protocol 2020.054). Written informed consent was signed by all participants.

Analytical assessments were conducted on serum samples obtained following an overnight fasting period. Biochemical analyses to evaluate carbohydrate, lipid, hepatic, and inflammatory profiles were performed using established methods (Unamuno et al. 2021). Insulin resistance and sensitivity were calculated using the homeostasis model assessment (HOMA) and quantitative insulin sensitivity check index (QUICKI) indices, respectively. Leptin levels were determined using a double-antibody radioimmunoassay (RIA) method (Merck Millipore, Billerca, MA, USA).

### Experimental animal model

Four-week-old male Wistar rats (n = 72) were fed ad libitum during 4 months with a normal diet (ND, n = 10) (diet 2014S, 12.1 kJ/g, Harlan, Teklad Global Diets, Harlan Laboratories Inc., Barcelona, Spain) or a high-fat diet (HFD, n = 62) (diet F3282, 23.0 kJ/g, Bio-Serv, Frenchtown, NJ, USA). Experimental animals were housed in individual cages and maintained under pathogen-free conditions. The laboratory environment was carefully controlled, with a temperature set at 22 ± 2 °C and relative humidity maintained at 50 ± 10%. Additionally, the rats were kept on a 12:12 h light–dark cycle, with lights on at 08:00 am. Body weight and food intake were registered weekly. Rats with DIO were randomized to be submitted to sham surgery (n = 13), single anastomosis duodeno-ileal bypass with sleeve gastrectomy (SADI-S) (n = 7), sleeve gastrectomy (SG) (n = 10) and pair-fed (n = 22) to the amount of food eaten by animals undergoing each type of bariatric surgery in order to differentiate possible effects due to a decreased food intake. All procedures were performed as previously described (Becerril et al. 2024). Six weeks after the surgical and caloric interventions, experimental animals were fasted for 6 h and sacrificed. The epididymal AT (EWAT) was carefully dissected out, weighted and stored at −80 ºC for RNA studies. Experimental processes followed the European Guidelines for the Care and Use of Laboratory Animals (directive 2010/63/EU) and were approved by the Ethical Committee for Animal Experimentation of the University of Navarra (2019.089).

### Gene expression levels

TRIzol^®^ Reagent (Invitrogen, Carlsbad, CA, USA) and the RNeasy Lipid Mini Kit (Qiagen, Maryland, MD, USA) were used for RNA isolation as previously described. Subsequently, a treatment with DNase I (RNase Free DNase set, Qiagen) was performed to remove a possible DNA contamination. RNA was reverse transcribed using M-MLV reverse transcriptase (Invitrogen) and random hexamers (Invitrogen). Transcript levels of caveolin-1 (*CAV1*), interleukin (*IL*)-*1B*, *IL8*, *IL18*, leucine rich repeat containing 8 VRAC subunit A (*LRCC8A*/*SWELL1*), nucleotide-binding oligomerization domain, leucine-rich repeat, and pyrin (*NLRP*)-*3*, *NLRP6*, *PIEZO1* and sirtuin-1 (*SIRT1*) were quantified using Real-Time PCR (7300 Real-Time PCR System, Applied Biosystems, Foster City, CA, USA). Primers and probes design (Table S1) was performed using the Primer Express 2.0 software (Applied Biosystems) with the primer forward or the probes comprising the ends of two exons to prevent potential genomic DNA amplification (Merck, Darmstadt, Germany). The cDNA was then amplified using the TaqMan^®^ Universal PCR Master Mix (Applied Biosystems) as previously reported (Frühbeck et al. 2022). The *18S* rRNA (Applied Biosystems) served as endogenous control gene for relative quantification using the ΔΔCt formula.

### Histological analysis

Immunohistochemistry staining of PIEZO1 was conducted on sections of formalin-fixed paraffin-embedded VAT. VAT Sections (6 µm) from NW individuals and people with obesity from both groups (NG and T2D), were incubated overnight at 4 °C with rabbit monoclonal anti-PIEZO1 (PA5-106296, Thermo Fisher Scientific, Waltham, MA, USA), diluted 1:50 in Tris-buffered saline (TBS, Merck). After washing, slides were incubated with anti-rabbit secondary antibodies conjugated with DAKO Real EnVision™ horseradish peroxidase (DakoCytomation, Glostrup, Denmark) for 1 h at RT. The peroxidase reaction was visualized using a 0.5 mg/mL diaminobenzidine/0.03% H_2_O_2_ solution diluted in 50 mmol/L Tris–HCl, pH 7.36, and Harris hematoxylin solution (Sigma) as counterstaining. To examine the localization patterns of PIEZO1, the Zeiss Axiovert CFL light microscope (Zeiss, Göttingen, Germany) was used. Negative control slides without primary antibody were included in the process to assess non-specific staining. ImageJ analysis software (NIH, USA, https://imagej.net/ij/download.html) was used for quantitative evaluation.

### Cell cultures

Stromal vascular fraction cells (SVFC) were isolated from VAT. Cells were seeded at a density of 2 × 10^5^ cells per well and cultured in adipocyte medium, following previously established protocols. Adipocytes reached approximately 70–75% differentiation (as assessed by morphological changes) by the eighth day of differentiation. Adipocyte-conditioned medium (ACM) was obtained by collecting supernatant from differentiated adipocytes, which was then centrifuged and diluted to 20% and 40% concentrations in RPMI-1640 medium (Thermo Fisher Scientific, Waltham, MA, USA). In addition, mature adipocytes were treated with increasing concentrations of LPS (10, 100 and 1000 ng/mL) (Merck), interferon-γ (IFN-γ) (20, 50 and 100 ng/mL) (R & D Systems, Minneapolis, MN, USA), insulin (1, 10 and 100 ng/mL) (Merck), and transforming growth factor β (TGF-β) (1, 10 and 20 ng/mL) (R & D Systems) at 37 °C for 24 h.

The THP-1 monocyte cell line (TIB-202TM) was obtained from the ATCC^®^ (Middlesex, UK) and cultured following as previously reported (Frühbeck et al. 2022). To induce differentiation of THP-1 monocytes into macrophage-like cells, THP-1 cells were treated with 25 ng/mL phorbol 12-myristate 13-acetate (PMA, Merck) and incubated for 24 h (Baxter et al. 2020). Subsequently, the PMA was removed by washing, and cells were maintained for 24 h in fresh RPMI-1640 medium before the exposure to ACM at 20% and 40%.

### Adipose tissue compression assay

VAT explants were obtained from patients with obesity during RYGB surgery. Briefly, AT was dissected to eliminate blood vessels and connective tissue and minced in small fragments with sterile scissors. After, fragments were washed with PBS buffer, rinsed with adipocyte culture medium and distributed into 6-well plates (Thalmann et al. 2008). Custom compression adapters were built in order to perform the mechanical compression testing of VAT explants to allow the culture medium to distribute throughout the wells and not create areas of hypoxia in the tissue explants. The 3D design of the adapters was made using the Autodesk Fusion 360 software (Autodesk Inc, CA, USA) and were 3D printed using a FDM Lulzbot TAZ 6 3D printer (Lulzbot, N.D, USA) and polylactic acid (PLA) (Smart materials 3D, Jaén, Spain) as the fused material. Compression forces were applied with precision weights of class M1 made of A3 steel of 100 and 200 g (Daselab SL, Valencia, Spain) corresponding to 2 and 4 kPa, respectively. Explants were incubated at 37 °C for 24 h.

### Statistical analysis

G*Power 3.1.9.4 program (Franz Faul, University of Kiel, Germany) was used for sample size estimation with preliminary data obtained in our own experience (Unamuno et al. 2021). Data are presented as mean ± standard error of the mean (SEM). One-way ANOVA followed by Tukey’s post hoc tests and two-tailed unpaired Student’s* t* tests were applied to assess differences in gene expression levels as appropriate. Correlations between two variables were computed by Pearson’s correlation coefficients. Statistical analysis was performed using SPSS v23 (Chicago, IL, USA) and GraphPad Prism v8.3 (GraphPad Software, Inc., San Diego, CA, USA) and *P* < 0.05 was considered statistically significant.

## Results

### Increased expression of *PIEZO1* in VAT in obesity and obesity-associated T2D

**Fig. 1:**
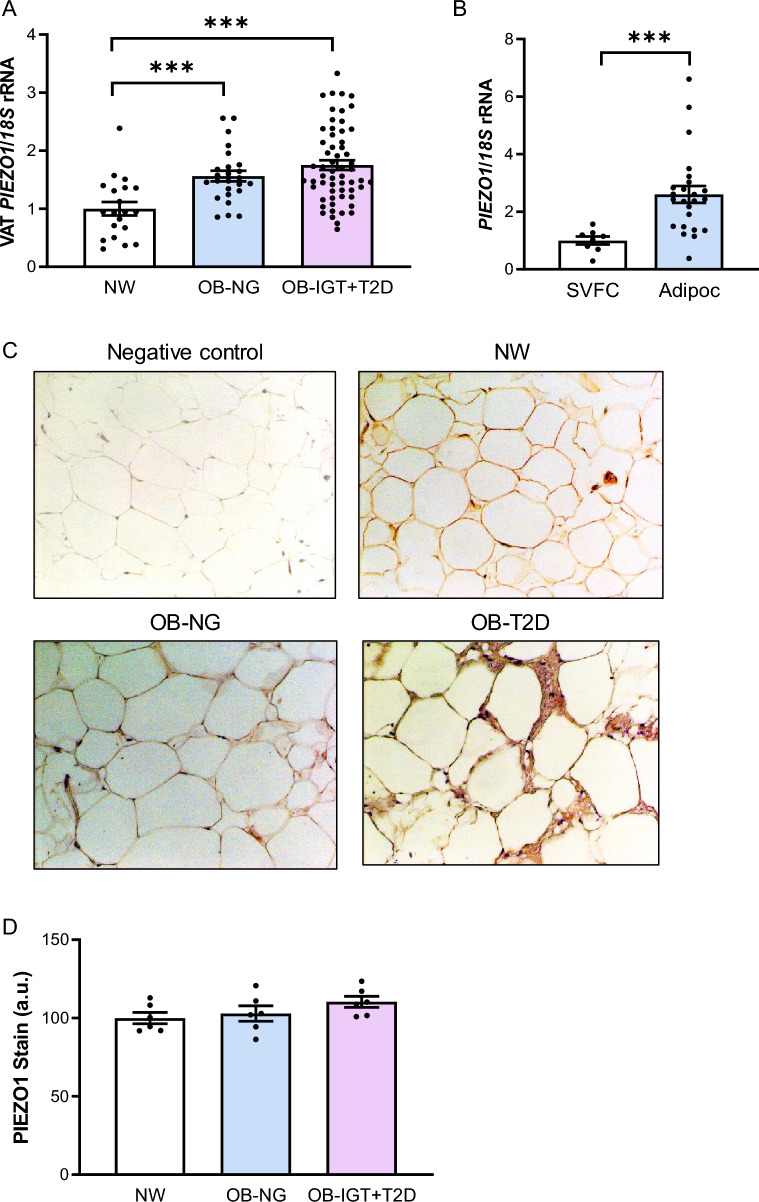
Gene expression levels of PIEZO1 in visceral adipose tissue (VAT). **A** Bar graphs show the mRNA levels of *PIEZO1* in VAT samples from volunteers with normal weight (NW) and people with obesity and normoglycemia (OB-NG) and with obesity-associated type 2 diabetes (OB-T2D). **B** Gene expression levels of *PIEZO1* in adipocytes and stroma-vascular fraction cells (SVFC) obtained from patients with obesity. **C** Representative immunohistochemistry and (**D**) quantification (n = 6) for PIEZO1 in VAT from volunteers with NW, with OB-NG and with OB-T2D (magnification 100×, scale bar 50 µm). Differences between groups were analyzed by one-way ANOVA followed by Tukey’s tests as well as by unpaired two-tailed Student’s *t* test. ^***^P < 0.001

Anthropometric and biochemical data of the patients involved in the study are summarized in Table 1. Results showed increased (*P* < 0.05) transcript levels of *PIEZO1* in patients with obesity and obesity-associated T2D in VAT (Fig. 1A). In this line, a significant association between *PIEZO1* mRNA levels and weight (r = 0.26, *P* = 0.010), BMI (r = 0.32, *P* = 0.002) and BF (r = 0.24, *P* = 0.024) was observed. No sexual dimorphism was found regarding *PIEZO1* mRNA levels (female: 1.00 ± 0.05 *vs* male: 1.03 ± 0.07 au; *P* = 0.710). A strong upregulation (*P* < 0.001) of gene expression levels of *PIEZO1* in adipocytes compared to the SVFC was also found (Fig. 1B). In addition, we verified the presence of PIEZO1 in sections of human VAT by immunohistochemistry analysis (Fig. 1C) finding that immunoreactivity for PIEZO1 was notably increased in the membrane of the adipocytes compared to the SVFC. Although we detected an increase in the amount of total immunostaining of PIEZO1 in VAT biopsies obtained from patients with obesity and T2D compared to that of volunteers with NW and with obesity and NG, differences were not statistically significant (Fig. 1D).

**Table 1:** Anthropometric and biochemical characteristics of subjects included in the study

	NW	OB-NG	OB-IGT + T2D
n (male, female)	20 (7, 13)	25 (10, 15)	55 (24, 31)
Age (years)	44 ± 4	40 ± 2	46 ± 1
BMI (kg/m^2^)	22.5 ± 0.5	42.6 ± 1.3^***^	45.2 ± 0.8^***^
Body fat (%)	22.1 ± 1.9	53.3 ± 1.5^***^	50.8 ± 0.9^***^
Waist circumference (cm)	76 ± 3	119 ± 3^***^	134 ± 2^***,††^
WHR	0.80 ± 0.02	0.90 ± 0.02^*^	1.01 ± 0.01^***,†††^
WHtR	0.46 ± 0.01	0.72 ± 0.02^***^	0.80 ± 0.01^***,†††^
Fasting glucose (mg/dL)	88 ± 3	89 ± 2	129 ± 6^***,†††^
2 h OGTT glucose (mg/dL)	–	118 ± 4	194 ± 8^†††^
Fasting insulin (µU/mL)	6.8 ± 1.0	15.0 ± 1.7	29.8 ± 5.2
2 h OGTT insulin (µU/mL)	–	103.3 ± 1.3	150.0 ± 1.9
HOMA	1.5 ± 0.4	3.4 ± 0.4^*^	9.1 ± 1.6^*,†^
QUICKI	0.375 ± 0.016	0.325 ± 0.006^***^	0.291 ± 0.005^***,†††^
HbA1c (%)	–	5.57 ± 0.09	7.46 ± 0.29^†††^
Triglycerides (mg/dL)	84 ± 11	97 ± 8	143 ± 7^***,††^
Cholesterol (mg/dL)	169 ± 21	195 ± 7	186 ± 4
LDL-cholesterol (mg/dL)	113 ± 16	124 ± 6^*^	113 ± 3
HDL-cholesterol (mg/dL)	74 ± 7	53 ± 2^***^	45 ± 2^***^
Leptin (ng/mL)	8.2 ± 1.8	55.8 ± 4.8^***^	45.1 ± 4.2^***^
Leucocytes (× 10^6^)	6.76 ± 0.33	7.58 ± 0.41	7.86 ± 0.35
CRP (mg/L)	1.8 ± 0.2	10.4 ± 1.8^**^	8.0 ± 0.9^**^
Homocysteine (µmol/L)	7.8 ± 1.7	8.5 ± 0.5	11.4 ± 0.8
AST (U/L)	13 ± 1	18 ± 2	22 ± 2
ALT (U/L)	9 ± 2	26 ± 5^*^	30 ± 3^**^
AST/ALT	1.73 ± 0.20	0.85 ± 0.04^***^	0.76 ± 0.03^***^
γ-GT (U/L)	11 ± 2	27 ± 5	38 ± 6

*ALT* alanine aminotransferase, *AST* aspartate aminotransferase, *BMI* body mass index, *CRP* C-reactive protein, *γ-GT* γ-glutamyltransferase, *HBA1c* glycated haemoglobin, *HOMA* homeostatic model assessment, *IGT* impaired glucose tolerance, *NG* normoglycemia, *NW* normal weight, *OB* obesity, *OGTT* oral glucose tolerance test, *QUICKI* quantitative insulin sensitivity check index, *T2D* type 2 diabetes, *WHR* waist-to-hip ratio, *WHtR* waist-to-height ratio

Data are mean ± SEM. Differences between groups were analyzed by one-way ANOVA followed by Tukey’s post hoc tests or by unpaired two-tailed Student’s *t* tests, where appropriate. ^*^P < 0.05, ^**^P < 0.01 and ^***^P < 0.001 vs NW. ^†^*P* < 0.05, ^††^*P* < 0.01 and ^†††^*P* < 0.01 vs OB-NG

### *PIEZO1* is associated with *SWELL1* and key markers of inflammation in VAT

**Fig. 2:**
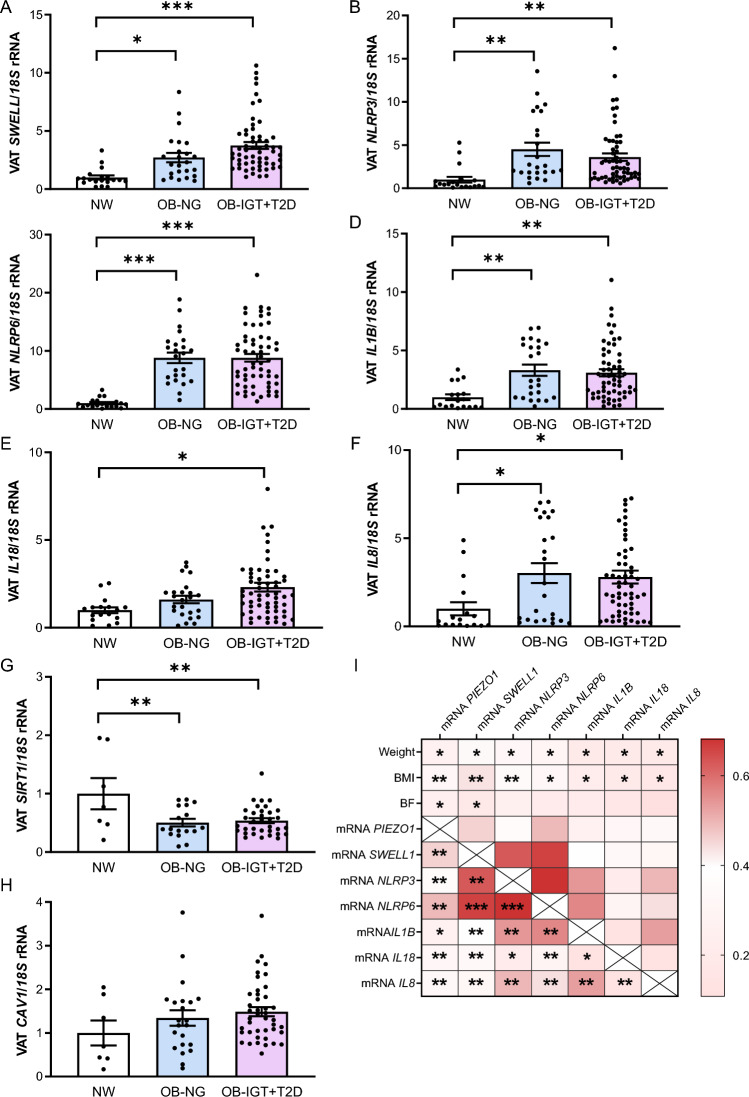
Impact of obesity and obesity-associated T2D on VAT expression levels of the key inflammation-related factors and their association with *PIEZO1*. Bar graphs show the gene expression levels of (**A**) *LRCC8A*/*SWELL1*, (**B**) *NLRP3*, (**C**) *NLRP6*, (**D**) *IL1B*, (**E**) *IL18*, (**F**) *IL8*, (**G**) *SIRT1* and (**H**) *CAV1* in VAT samples from volunteers with normal weight (NW) and people with obesity and normoglycemia (OB-NG) and with obesity-associated type 2 diabetes (OB-T2D). **I** Heatmap of the associations between gene expression levels of *PIEZO1* and inflammation-related factors in VAT. Differences between groups were analyzed by one-way ANOVA followed by Tukey’s tests. ^*^P < 0.05, ^**^P < 0.01 and ^***^P < 0.001. BMI, body mass index, BF body fat, *LRCC8A*/*SWELL1* leucine rich repeat containing 8 VRAC subunit A, *CAV1* caveolin-1, *NLRP* nucleotide-binding oligomerization domain, leucine-rich repeat and pyrin, *IL* interleukin, *SIRT1* sirtuin 1

While PIEZO1 mainly responds to mechanical forces, SWELL1, another anion channel, is primarily involved in controlling cell volume constituting a crucial component in the regulation of adipocyte size (Zhang et al. 2017). Activation of PIEZO1 involves changes in membrane tension and in cell volume, probably interacting or cooperating with other VRAC, including SWELL1. Upregulated mRNA levels of *SWELL1* were found in patients with obesity and NG (*P* < 0.05) as well as in those with obesity and T2D (*P* < 0.001) (Fig. 2A). Interestingly, *PIEZO1* and *SWELL1* expression levels were positively associated (*P* < 0.01) (Fig. 2I). PIEZO1 responds to different mechanical stresses that trigger inflammation. For this, we analyzed its relationship with mediators of inflammation in VAT. We detected increased mRNA levels of *NLPR3* (*P* = 0.002), *NLRP6* (*P* < 0.001) and its effectors *IL1B* (*P* = 0.002), *IL8* (*P* = 0.047) and *IL18* (*P* = 0.006) in VAT of patients with obesity and with obesity and T2D (Fig. 2B–F). Interestingly, a positive correlation of *PIEZO1* with all measured inflammatory markers was found (Fig. 2I). The associations between the mRNA levels of *PIEZO1* and *SWELL1* as well as with the inflammatory markers *IL1B* and *IL18* remained statistically significant (*P* < 0.05) after controlling for body weight and body fat. SIRT1 plays multifaceted roles in obesity including the regulation of inflammation by inhibiting nuclear factor κ-B (NF-κB), thereby reducing inflammatory responses and improving insulin sensitivity (Liang et al. 2009). We found decreased (*P* < 0.05) mRNA levels of *SIRT1* in both groups of individuals with obesity (Fig. 2G). SIRT1 can be modulated by changes in cellular calcium levels, suggesting that the ionic changes induced by PIEZO1 could indirectly affect SIRT1 activity. However, no statistically significant association between *PIEZO1* and *SIRT1* mRNA levels was detected. Since mechanical stretch activates PIEZO1 in caveolae, the expression of *CAV1* was analyzed and, although a tendency towards an increase in patients with obesity was detected, differences did not reach statistical significance (Fig. 2H). No association between transcript levels of *PIEZO1* and *CAV1* was found.

### Expression of *Piezo1* in AT from HFD-fed rats decreased after bariatric surgery

**Fig. 3:**
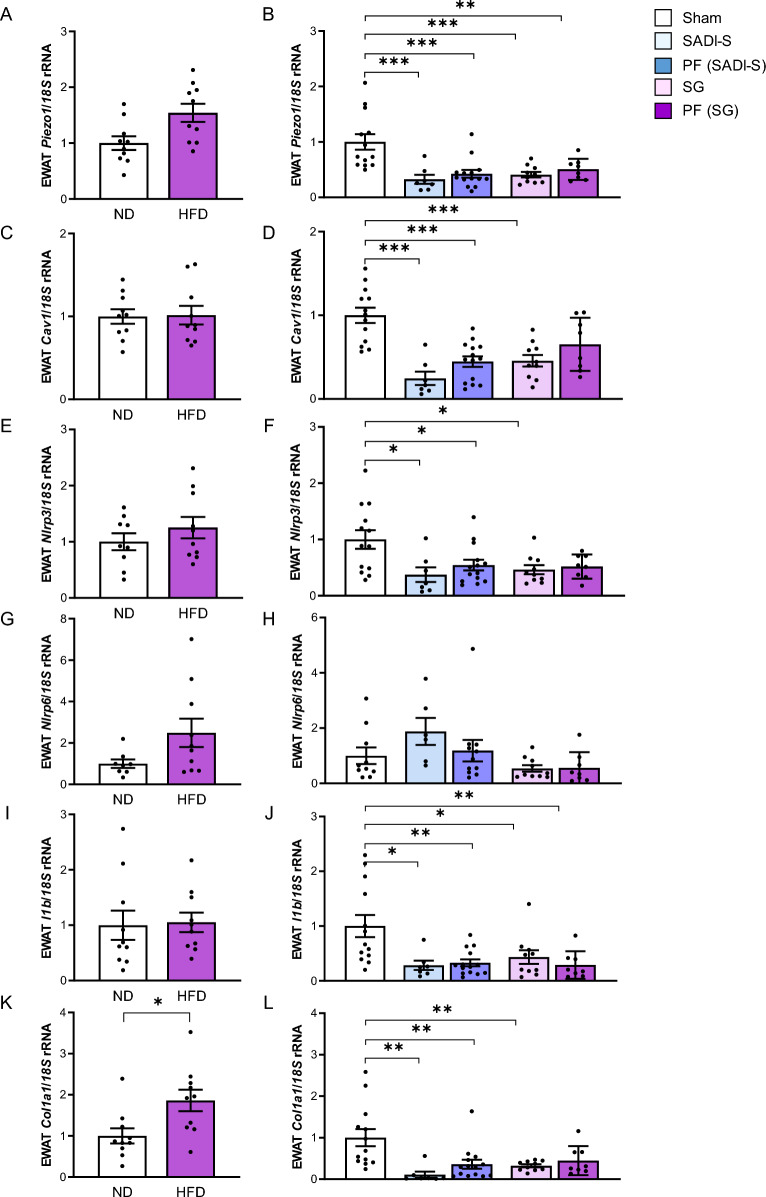
Gene expression levels of *Piezo1* (**A**), *Cav1* (**C**), *Nlrp3* (**E**), *Nlrp6* (**G**), *Il1b* (**I**) and *Col11a1* (**K**) in the epididymal adipose tissue (EWAT) from rats fed a normal diet (ND) or a high-fat diet (HFD) and (**B, D, F, H, J, L**) after being submitted to sham surgery, single anastomosis duodeno-ileal bypass with sleeve gastrectomy (SADI-S), sleeve gastrectomy (SG) and pair-fed (PF). Differences between groups were analyzed by unpaired two-tailed Student’s *t* test as well as by one-way ANOVA followed by Tukey’s tests. ^*^P < 0.05, ^**^P < 0.01 and ^***^P < 0.001. *Cav1*, caveolin 1; *Col1a1*, collagen 1a1; *Il1b*, interleukin-1β; *Nlrp*, nucleotide-binding oligomerization domain, leucine-rich repeat and pyrin

The HFD increased body (ND-fed rats 508 ± 12 g vs HFD-fed rats 548 ± 17 g, *P* < 0.05) and EWAT (ND-fed rats 5.8 ± 0.4 g vs HFD-fed rats 9.9 ± 1.0 g, *P* < 0.05) weights (Supplemental Fig. 1). The HFD also promoted the development of insulin resistance as confirmed by higher glucose levels (ND-fed rats 98 ± 2 mg/dL vs HFD-fed rats 117 ± 3 mg/dL, *P* < 0.001) and a lower QUICKI index (ND-fed rats 0.368 ± 0.028 vs HFD-fed rats 0.341 ± 0.023 *P* < 0.05) (Supplemental Fig. 1). In line with findings in human samples, we found a tendency towards increased transcript levels of *Piezo1* in the EWAT obtained from rats with DIO although differences were not statistically significant (Fig. 3A). Importantly, mRNA levels of *Piezo1* were associated with body weight (r = 0.30,* P* = 0.010), EWAT weight (r = 0.43,* P* < 0.001) and glucose levels (r = 0.35,* P* = 0.003). We also detected increased (*P* < 0.05) gene expression levels of *Col1a1* in the EWAT from rats under a HFD (Fig. 3). Although tendency towards an increase in the expression levels of the inflammatory markers *Nlrp3*, *Nlrp6* and *Il1b* was detected, differences did not reach statistical significance (Fig. 3). No differences were found regarding mRNA levels of *Cav1* (Fig. 3).

Both types of bariatric surgical procedures induced body and EWAT weight loss (*P* < 0.01) compared to sham counterparts, being higher in the animals submitted to SADI-S. In addition, the absolute food intake of SADI-S-operated rats was significantly lower (*P* < 0.001) than that of sham- and SG-operated animals. We confirmed a strong downregulation (*P* < 0.001) of gene expression levels of *Piezo1* after both surgeries compared to rats submitted to simulated surgery (Fig. 3B). Additionally, pair-fed animals also showed reduced (*P* < 0.001) mRNA levels of *Piezo1* (Fig. 3B). As expected, both types of surgeries significantly downregulated (*P* < 0.01) the gene expression levels of *Nlrp3, Il1b, Col1a1* and *Cav1* in the EWAT from the experimental animals (Fig. 3). In addition, gene expression levels of *Piezo1* were significantly associated with mRNA levels of *Il1b* and *Col1a1*.

### Static compression boosts an upregulation of *PIEZO1* in VAT explants together with a strong inflammation

**Fig. 4:**
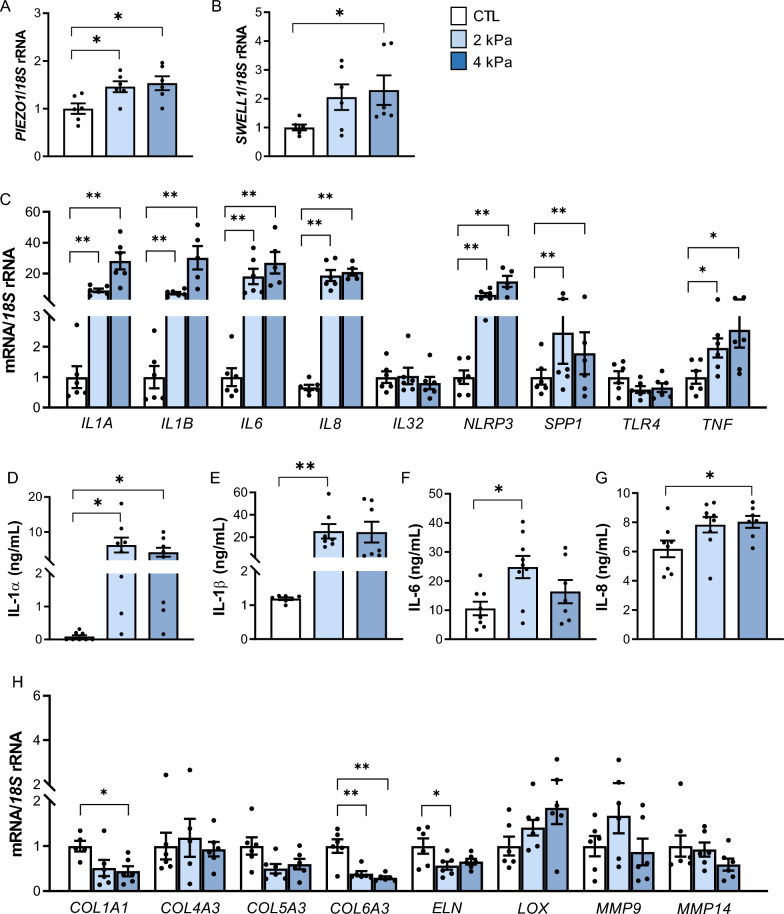
Effect of static mechanical compression on VAT explants. Gene expression levels of (**A**) *PIEZO1*, (**B**) *SWELL1*, (**C**) key inflammatory mediators and (**H**) extracellular matrix-related factors after 2 and 4 kPa of mechanical compression for 24 h. Secreted concentrations of the crucial inflammatory markers (**D**) IL-1α, (**E**) IL-1β, (**F**) IL-6 and (**G**) IL-8 in the culture media of human VAT explants after static compression for 24 h. Values are the mean ± SEM. Differences between groups were analyzed by one-way ANOVA followed by Tukey’s tests. ^*^P < 0.05 and ^**^P < 0.01. *COL* collagen; *ELN* elastin, *IL* interleukin, *LOX* lysyl oxidase, *LRCC8A*/*SWELL1* leucine rich repeat containing 8 VRAC subunit A, *MMP* matrix metalloproteinase, *NLRP* nucleotide-binding oligomerization domain, leucine-rich repeat and pyrin, *SPP1* osteopontin, *TLR4* toll-like receptor 4, *TNF* tumour necreosis factor-α

Considering *PIEZO1* is induced by mechanical forces, we next analyzed its gene expression levels as well as the transcript levels of *SWELL1* and relevant inflammation-related genes after the static compression of VAT explants. Importantly, we found an upregulation of *PIEZO1* (*P* = 0.017) and *SWELL1* (*P* = 0.042) after VAT compression (Fig. 4A, B). Furthermore, a strong increase in the gene expression levels of *IL1A* (*P* < 0.001), *IL1B* (*P* < 0.001), *IL6* (*P* < 0.001), *IL8* (*P* < 0.001), *NLRP3* (*P* < 0.001), *SPP1* (*P* < 0.01) and *TNF* (*P* < 0.05) was observed (Fig. 4C). To examine the impact of compression on the inflammatory response, the levels of the inflammatory mediators released into the culture medium were determined. Compression promoted an increase of IL-1α (*P* < 0.01), IL-1β (*P* < 0.001), IL-6 (*P* < 0.05), and IL-18 (*P* < 0.05) concentrations into the culture medium (Fig. 4D–G). Since increased inflammation has been extensively associated with fibrosis, our next objective was to explore the expression levels of different ECM proteins (Fig. 4H). Interestingly, *COL1A* (*P* < 0.05), *COL6A3* (*P* < 0.001), and *ELN* (*P* < 0.05) mRNA levels decreased after mechanical stretch. No differences were found in the regulation of other collagens (*COL4A3* and *COL5A3*), matrix metalloproteinases (*MMP9*, and *MMP14*) and *LOX*.

### *PIEZO1* is regulated by inflammation in visceral adipocytes

**Fig. 5:**
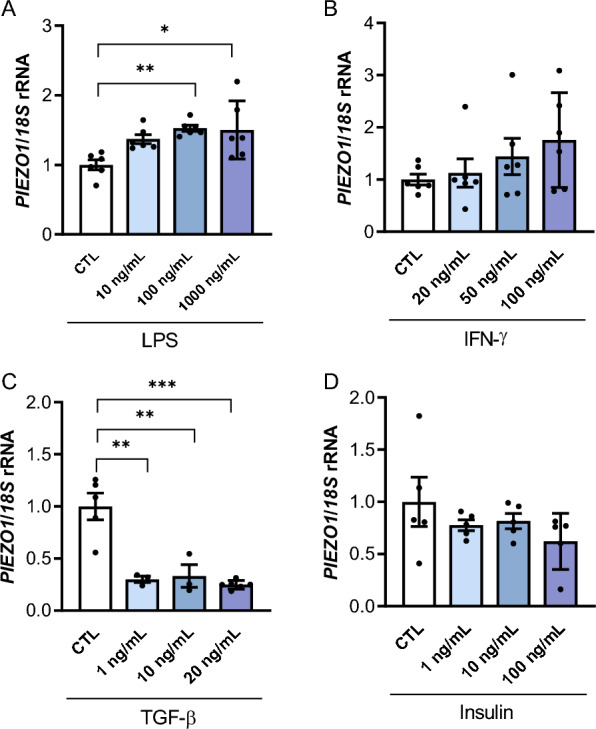
Gene expression levels of *PIEZO1* in human visceral adipocytes treated with different concentrations of (**A**) lipopolysaccharide (LPS), (**B**) interferon-γ (INF-γ, (**C**) transforming growth factor-β (TGF-β) and (**D**) insulin during 24 h. Values are the mean ± SEM. Differences between groups were analyzed by one-way ANOVA followed by Tukey’s tests. ^*^P < 0.05, ^**^P < 0.01 and ^***^P < 0.001

As shown in Fig. 5A LPS (*P* < 0.05) upregulated the mRNA levels of *PIEZO1* in human adipocytes. A tendency towards increased expression was detected after the stimulation with IFN-γ but differences did not reach statistical significance (Fig. 5B). Oppositely, TGF-β, the master driver of fibrosis, downregulated *PIEZO1* transcript levels (*P* < 0.01) (Fig. 5C) while no differences were found after insulin treatment (Fig. 5D).

### Adipocyte-M1 macrophage crosstalk in the expression levels of *PIEZO1*

**Fig. 6:**
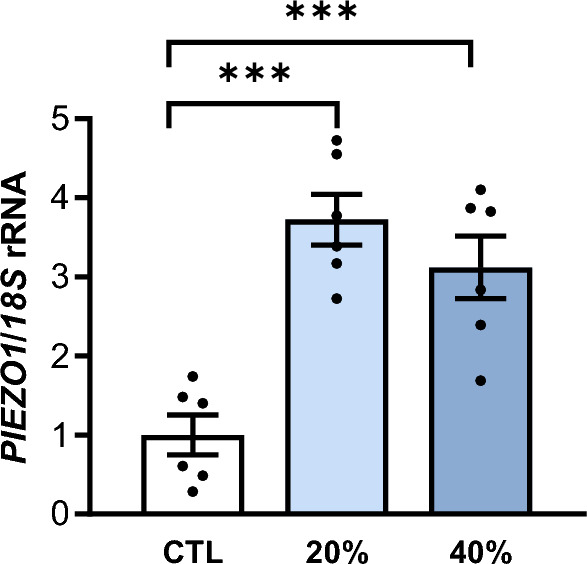
Effect of adipocyte-conditioned media (ACM) in THP-1-derived macrophages. Bar graphs show the effect of ACM (20 and 40%) from subjects with obesity incubated for 24 h on the transcript levels of PIEZO1. Differences between groups were analyzed by one-way ANOVA followed by Tukey’s tests. ^***^P < 0.001

Since *PIEZO1* is upregulated in the VAT from individuals with obesity and with obesity and T2D, mainly in adipocytes, we further analyzed the adipocyte-macrophage crosstalk by studying the effect of the adipocyte secretome obtained from patients with obesity in the expression of *PIEZO1* in THP-1-derived macrophages. A robust upregulation (*P* < 0.001) of the gene expression levels of *PIEZO1* in M1 macrophages after the treatment with the ACM was observed (Fig. 6).

## Discussion

Emerging evidence suggests the important role of PIEZO1 as a mechanosensitive ion channel in AT biology, energy metabolism, and the development of obesity-related metabolic disorders by impacting on AT mechanotransduction and inflammation. The main findings of our study are that: (i) obesity and T2D increase gene expression levels of *PIEZO1* in VAT being associated with *SWELL1* and key markers of inflammation, (ii) a static compression of VAT explants promotes an upregulation of *PIEZO1* together with a robust inflammatory response, and (iii) the secretome of adipocytes from patients with obesity upregulates *PIEZO1* levels in THP-1-derived macrophages. In addition, we also confirmed decreased expression levels of *Piezo1* in the EWAT from rats with DIO submitted to two different types of bariatric surgery, SADI-S and SG.

Adipocytes sense mechanical forces from their microenvironment to regulate their homeostasis and to coordinate their behavior at local and systemic levels. These forces depend on the physical properties of the ECM, the neighboring cells, and the fluids around cells (Romani et al. 2021). In the context of chronic obesity, the disrupted balance between the synthesis and degradation of the ECM components results in their excessive accumulation, diminishing ECM plasticity and functionality and, consequently, a cascade of maladaptive responses leading to inflammation and fibrosis, being the adipocytes under a constant mechanical pressure (Gliniak et al. 2023). PIEZO1 has emerged as a key mechanoreceptor highly expressed in mechanical stressed tissues and, therefore, in AT. Our data showed that gene expression levels of *PIEZO1* were increased in VAT from patients with obesity and obesity-associated T2D, being adipocytes the main responsible source for *PIEZO1* expression. In line with our findings, upregulated *Piezo1* levels in adipocytes but not in the SVF of AT from mice with obesity has been described (Zhao et al. 2019), strengthening the involvement of PIEZO1 in the regulation of biological functions of adipocytes. Consistently, Zhao et al. (2019) described that AT exhibited the highest PIEZO1 expression among 37 human and 18 mouse tissues. A crucial role of PIEZO1 in β-cell function has been suggested since its inhibitor GsMTx4 reduced glucose-induced insulin secretion in human islets (Ye et al. 2022) and its agonist Yoda-1 induced insulin release from β-cell lines and mouse pancreatic islets (Deivasikamani et al. 2019). Furthermore, PIEZO1 exhibited increased expression levels in islets from both humans and rodents with T2D (Ye et al. 2022). Although the precise mechanisms linking PIEZO1 and insulin resistance are not fully understood, adipocyte hypertrophy may also be an important mediator. Mice lacking *Piezo1* in mature adipocytes showed an impaired differentiation of preadipocyte into mature adipocytes when summited to a HFD, leading to larger adipocytes and decreased insulin sensitivity (Wang et al. 2020). In contrast, *Piezo1* knockout mice had no significantly smaller adipocyte size but showed increased triglyceride levels in the liver (Zhao et al. 2019). This difference might be the consequence of compensatory mechanisms induced by long-term absence of *Piezo1* in the constitutive knockout model. By regulating ghrelin production, PIEZO1 has been also indirectly involved in obesity development. Patients with obesity exhibited a downregulation of PIEZO1 together with an upregulation of ghrelin in the X/A-like cells maybe contributing to overeating and obesity (Zhao et al. 2024). Importantly, we found decreased transcript levels of *Piezo1* in the EWAT from rats submitted to bariatric surgery and also in the pair-fed counterparts. We suggest that the reduced size of the adipocytes together with the improvements in insulin resistance and inflammation associated to weight loss achieved by surgery or caloric restriction may be the responsible for the reduction in *Piezo1* expression levels.

PIEZO1 also functions in combination with other depolarizing membrane proteins, including SWELL1 (Ye et al. 2022). We found increased *SWELL1* mRNA levels in both groups of patients with obesity as well as a positive association with *PIEZO1*. Reportedly, SWELL1 is activated in the context of adipocyte hypertrophy in obesity being required for maintaining insulin signaling and glucose homeostasis by enhancing insulin-PI3K-AKT2 signaling (Zhang et al. 2017; Xie et al. 2017). Mechanical stress can activate both PIEZO1 and SWELL1, suggesting a coordinated response to mechanical and osmotic stress with changes in cell volume activating PIEZO1 and the influx of Ca^2+^ through PIEZO1 influencing the activity of VRACs. In addition, the increase in intracellular Ca^2+^ controls inflammation by acting as a secondary messenger in different signaling pathways, including the activation of the inflammasomes (Lee et al. 2012). Two important components of the inflammasomes, *NLRP3* and *NLRP6* together with their mediators *IL1B* and *IL18* were upregulated in VAT of patients with obesity and obesity-associated T2D and were significantly associated with *PIEZO1*. It suggests that obesity-associated mechanical stress sensed by PIEZO1 leads to persistent Ca^2+^ influx, potentially contributing to sustained NLRP activation and chronic inflammation. Similar results have been found in intestinal epithelial cells suggesting that PIEZO1 activates the NLRP3 inflammasome and promotes intestinal inflammation (Liu et al. 2023). Apart from the inflammasome activation, PIEZO1 participates in inflammation through a great range of signaling pathways including the classic inflammation pathways JAK-STAT, NF-κB, IL-6 receptor family or AKT/mTOR, perfectly reviewed by Liu et al. (2022). Importantly, after the static compression of VAT explants we observed an increase in the mRNA levels of *PIEZO1* and *SWELL1* as well as in the expression and secretion of IL-1α, IL-1β, IL-6 and IL-8. Additionally, an upregulation of *NLRP3*, *TNF* and *SPP1/OPN* was detected. Although mechanical stretch induces significant changes in the ECM structure of adipose tissue, altering the organization of collagen fibers and leading to increased stiffness of the ECM (Blade et al. 2024), we found less impact on ECM-related genes after the static compression of AT explants. We propose that following compression, the AT rapidly responds with an inflammatory reaction, whereas a longer duration may be required to observe changes in the remodelling of the ECM. The decreased mRNA expression of *COL1A1* and *COL6A3* after mechanical compression found in our study may be attributed to a compensatory mechanism by which adipose tissue undergoes structural adaptation to reduce mechanical stress, where a reduction in the production of COL1A1 and COL6A3 may facilitate remodeling and enhance tissue plasticity. Additionally, the strong release of pro-inflammatory cytokines after compression might potentially inhibit collagen production. Finally, sustained mechanical stress may elevate the activity of matrix metalloproteinases, enzymes responsible for ECM degradation, which could further contribute to the observed reduction in collagen levels. Although a clear connection between the inflammatory response and fibrosis in the AT takes place, it is crucial to consider that inflammation and fibrosis are also regulated by distinct mechanisms (Gliniak et al. 2023; Frühbeck et al. 2001; Heinonen et al. 2014; McVicker and Bennett 2017).

Visceral adipose tissue in patients with obesity is characterized by a pronounced infiltration of macrophages, which contributes to a pro-inflammatory microenvironment (Weisberg et al. 2003). This accumulation of immune cells within the adipose tissue is associated with metabolic dysregulation and plays a central role in the pathogenesis of obesity-related complications (Sell et al. 2012). The function of Piezo1 in macrophages has been increasingly linked to the regulation of inflammatory responses. In this regard, Piezo1 activation in macrophages can influence processes such as phagocytosis, cytokine release, and immune cell migration, all of which are relevant in the microenvironment of adipose tissue in obesity (Tang et al. 2023). Macrophages lacking *Piezo1* exhibit reduced inflammation and enhanced wound healing responses (Tang et al. 2023). Moreover, after the stimulation with IFN-γ and LPS, macrophages exhibited increased *Piezo1* expression (Atcha et al. 2021) suggesting that Piezo1 acts as an important regulator of macrophage function and polarization (Tang et al. 2023). We showed that macrophages treated with the ACM obtained from patients with obesity also upregulated the expression of *PIEZO1*, establishing a potential role of adipocytes enhancing the mechanosensitive behavior of macrophages, potentially exacerbating the pro-inflammatory environment, as well as promoting macrophage polarization. Understanding the role of Piezo1 in macrophages could provide insights into how mechanosensitive pathways contribute to obesity-associated inflammation and metabolic dysregulation. In this line, we also demonstrated that LPS increased the expression of *PIEZO1* in adipocytes while no significant effect was observed for IFN-γ.

Since activated PIEZO1 exhibits varying effects depending on the types and conditions of mechanical stimulation and the environmental context (Wang et al. 2022), the study of PIEZO1 in different AT depots will help to understand its role in obesity and its associated comorbidities. Additionally, the measurement of protein expression levels may shed information about full protein functionality. While our group and others have shown the participation of PIEZO1 in stretching assays, the present study highlights the relevance of compression forces in the biology of AT promoting changes in its inflammatory profile through different mechanisms, including PIEZO1. In addition, dynamic compression assays will be of relevance to further clarify the impact of compression forces on AT and to determine the role of the mechanical sensors in obesity and its association with inflammation. The genetic characterization of PIEZO1 will also help in the development of precision medicine approaches (Portincasa and Frühbeck 2023).

**Fig. 7:**
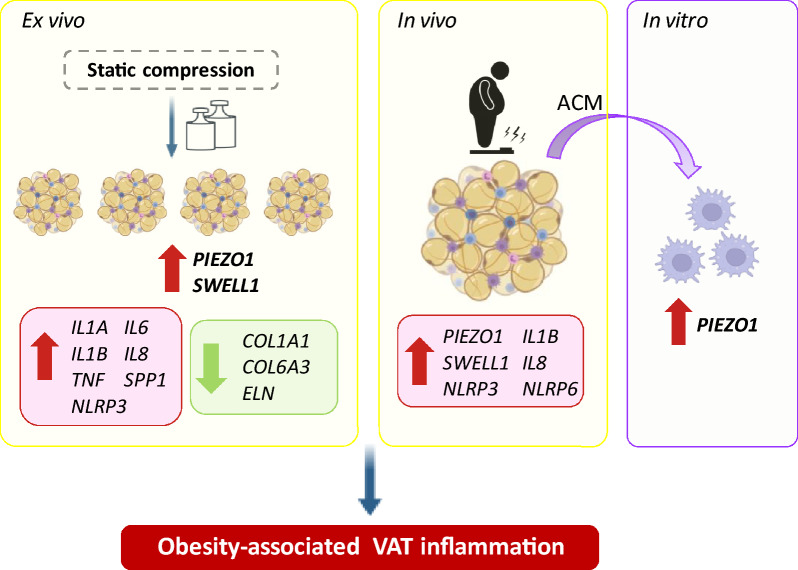
Static compression triggered an upregulation of *PIEZO1* and *SWELL1* expression levels in visceral adipose tissue (VAT) explants together with a strong increase in the expression and release of key inflammatory mediators (*IL1A*, *IL1B*, *IL6*, *IL8*, *NLRP3*, *SPP1*, *TNF*) and the downregulation of extracellular matrix factors (*COL1A1*, *COL6A3*, *ELN*). Obesity and obesity-associated T2D increased gene expression levels of *PIEZO1* in VAT mainly due to adipocytes. *SWELL1* and key markers of inflammation were also upregulated in VAT in obesity and T2D being significantly associated with *PIEZO1* levels. The treatment of THP-1-derived macrophages with the secretome of adipocytes from patients with obesity upregulated *PIEZO1* levels. *COL* collagen, *ELN* elastin, *IL* interleukin, *NLRP* nucleotide-binding oligomerization domain, leucine-rich repeat and pyrin, *SPP1* osteopontin, *SWELL1* leucine rich repeat containing 8 VRAC subunit A, *TNF* tumour necrosis factor-α

We found that the increased expression of *PIEZO1* in VAT in obesity and obesity-associated T2D is primarily attributable to adipocytes and is closely associated with *SWELL1* and inflammatory markers (Fig. 7). The interplay between mechanical forces and cell biology represents an interesting principle to understand how adipocyte biology is regulated by the tissue microenvironment, and how changes in tissue mechanics might contribute to the development of obesity-associated comorbidities. Further research in this area may uncover novel mechanisms underlying the involvement of PIEZO1 in obesity and its associated comorbidities.

## Supplementary Information

Supplementary Material 1.

## Data Availability

No datasets were generated or analysed during the current study.
