# Supplementary material for: Increased expression levels of PIEZO1 in visceral adipose tissue in obesity and type 2 diabetes are triggered by mechanical forces and are associated with inflammation
Source: Mol Med. 2024 Dec 20;30:255. doi: 10.1186/s10020-024-01008-1 (PMC11660983; doi:10.1186/s10020-024-01008-1)
Supplement: Supplementary file 1 — Supplementary Material 1. [file 10020_2024_1008_MOESM1_ESM.docx]

**Supplemental Table 1. Sequences of the primers and TaqMan^®^ probes.**

| **Gene (GenBank accession)** | | **Oligonucleotide sequence (5’-3’)** | | |
| --- | --- | --- | --- | --- |
| *CAV1* (NM_001753) | |  | | |
| Forward | | AACGATGACGTGGTCAAGATTG | | |
| Reverse | | TCCAAATGCCGTCAAAACTGT | | |
| TaqMan^®^ Probe | | FAM-TTGAAGATGTGATTGCAGAACCAGAAGGGA-TAMRA | | |
| *COL1A1* (NM_000088.3) | |  | | |
| Forward | | CTCCCGGGCCTCAAGGTAT | | |
| Reverse | | TTGCTCCAGAGGGACCTTGTT | | |
| TaqMan^®^ Probe | | FAM-TCTTCCTGGCCCCTCTGGTGAACCT-TAMRA | | |
| *COL4A3* (NM_000091.4) | |  | | |
| Forward | | AGTGGATTGCCAGGATTTTCTG | | |
| Reverse | | TGGTACACCGACCAGTCCGTAA | | |
| TaqMan^®^ Probe | | FAM-CCAGGCACCCCAGGCAATACCG-TAMRA | | |
| *COL5A3* (NM_015719.4) | |  | | |
| Forward | | GAACAAGGAAATTTGGACCTCAAG | | |
| Reverse | | GATTTGGAGCTGGAGTCTCTGTCT | | |
| TaqMan^®^ Probe | | FAM-TCCTGACTCCGCAGAGAACCAGACCTC-TAMRA | | |
| *COL6A3* (NM_004369.3) | |  | | |
| Forward | | GACGGAGATCTGGCTGATTTACA | | |
| Reverse | | AGATGCATTAGCCGCTCCAA | | |
| TaqMan^®^ Probe | | FAM-AGAACCTCCGCCAAGAAGGAGTCCGT-TAMRA | | |
| *ELN* (NM_000501.4) | |  | | |
| Forward | | TGGAGGAGTGGCAGCAAGA | | |
| Reverse | | CTTCCGGCCACAAGCTTTC | | |
| TaqMan^®^ Probe | | FAM-TCGGATTGTCTCCCATTTTCCCAGGT-TAMRA | | |
| *IL1A* (NM_000575) | |  | |  |
| Forward | | GTTCTGAAGAAGAGACGGTTGAGTTT | |  |
| Reverse | | AAGTTGTATTTCACATTGCTCAGGAA | |  |
| TaqMan^®^ Probe | | FAM-CATCGCCAATGACTCAGAGGAAGAAATCA-TAMRA | |  |
| *IL1B* (NM_000576) | |  | | |
| Forward | | CAGTGGCAATGAGGATGACTTG | | |
| Reverse | | GTAGTGGTGGTCGGAGATTCGTA | | |
| TaqMan^®^ Probe | | FAM-TGGCCCTAAACAGATGAAGTGCTCCTTCC-TAMRA | | |
| *IL6* (NM_000600) | |  | | |
| Forward | | GCCCTGAGAAAGGAGACATGTAAC | | |
| Reverse | | ATCCATCTTTTTCAGCCATCTTTG | | |
| TaqMan^®^ Probe | | FAM-AGGCACTGGCAGAAAACAACCTGAACC-TAMRA | | |
| *IL8* (NM_000584.3) | |  | | |
| Forward | | ACCTTTCCACCCCAAATTTATCA | | |
| Reverse | | TTCTCAGCCCTCTTCAAAAACTTC | | |
| TaqMan^®^ Probe | | FAM-CCACACTGCGCCAACACAGAAATTATTGTA-TAMRA | | |
| *IL32* (NM_001012631) | |  | |  |
| Forward | | GAGACAGTGGCGGCTTATTATGA | |  |
| Reverse | | GGCACCGTAATCCATCTCTTTCT | |  |
| TaqMan^®^ Probe | | FAM-CAGCACCCAGAGCTCACTCCTCTACTTGAA-TAMRA | |  |
| *LOX* (NM_001178102.2) | |  | |  |
| Forward | | ACACAGGACATCATGCGTATGC | |  |
| Reverse | | AACACCAGGCACTGATTTATCCA | |  |
| TaqMan^®^ Probe | | FAM-TCACCGTATTAGAAGGCAAAGCAAAACTCCC-TAMRA | |  |
| *LRRC8A* (NM_001127244.2) | |  | |  |
| Forward | | TACCTGGACCTCAGCCACAAC | |  |
| Reverse | | GAGCTTCCGGCACTGGAA | |  |
| TaqMan^®^ Probe | | FAM-CCAACCGGATCGAGACGCTCCC-TAMRA | |  |
| *MMP9* (NM_004994) | |  | |  |
| Forward | | GCCCGGACCAAGGATACAGT | |  |
| Reverse | | CCCCTCAGTGAAGCGGTACA | |  |
| TaqMan^®^ Probe | | FAM-ACGCGCTGGGCTTAGATCATTCCTCA-TAMRA | |  |
| *MMP14* (NM_004995.4) | |  | |  |
| Forward | | CGCCATCCAGGGTCTCAA | |  |
| Reverse | | TTGCGAATGGCCTCGTATG | |  |
| TaqMan^®^ Probe | | FAM-TCTGCATCCAGAATTAGACCCCCAAGGT-TAMRA | |  |
| *NLRP3* (NM_001079821.2) | |  | | |
| Forward | | AAGCTTCAGGTGTTGGAATTAGACA | | |
| Reverse | | GTTGCCCAGGCTCAGCTTT | | |
| TaqMan^®^ Probe | | FAM-CACACTGCTGCTGGGATCTTTCCACA-TAMRA | | |
| *NLRP6* (NM_138329.2) | |  | | |
| Forward | | TCCCTTCTTCATCCACTCTTTCAG | | |
| Reverse | | CAGACCGCGTCAGGGAGTT | | |
| TaqMan^®^ Probe | | FAM-CTGAGCAGCCTCACGCTGTCCCA-TAMRA | | |
| *PIEZO1* (NM_001142864.4) | |  | |  |
| Forward | | AACCCCGTGCCCAACTTTAT | |  |
| Reverse | | AGCACCAGCCAGAACAGGTATC | |  |
| TaqMan^®^ Probe | | FAM-CAGGTCCTACCTTGACATG-TAMRA | |  |
| *SPP1* (NM_000582) | |  | |  |
| Forward | | CATCCAGTACCCTGATGCTACAGA | |  |
| Reverse | | GGCCTTGTATGCACCATTCAA | |  |
| TaqMan^®^ Probe | | FAM-ACATCACCTCACACATGGAAAGCGAGGA-TAMRA | |  |
| *SIRT1* (NM_012238.5) | |  | |  |
| Forward | | ATAGAGCCTCACATGCAAGCTCTAG | |  |
| Reverse | | CTGTGCCAATCATAAGATGTTCCT | |  |
| TaqMan^®^ Probe | | FAM-ACTGGACTCCAAGGCCACGGATAGGTC-TAMRA | |  |
| *TLR4* (NM_003266) | |  | |  |
| Forward | | CTGCGTGGAGGTGGTTCCTA | |  |
| Reverse | | CAGGTCCAGGTTCTTGGTTGAG | |  |
| TaqMan^®^ Probe | | FAM-TTTCTACAAAATCCCCGACAACCTCCCCT-TAMRA | |  |
| *TNF* (NM_000594) | |  | | |
| Forward | | CCCCAGGGACCTCTCTCTAATC | | |
| Reverse | | ACATGGGCTACAGGCTTGTCA | | |
| TaqMan^®^ Probe | | FAM-CCTCTGGCCCAGGCAGTCAGATCAT-TAMRA | | |
| *rCav1* (NM_031556) | |  | |  |
| Forward | | ACGACGACGTGGTCAAGATTG | |  |
| Reverse | | AGACAGCAAGCGGTAAAACCAA | |  |
| TaqMan^®^ Probe | | FAM-TGCGGAACCAGAAGGGACACACAGTTT-TAMRA | |  |
| *rCol1a1* (NM_053304) | |  | |  |
| Forward | | TGTATCACCAGACGCAGAAGTCA | |  |
| Reverse | | GCTGATTTCTCATCATAGCCATAGG | |  |
| TaqMan^®^ Probe | | FAM-CCCCTGGTCTTGGAGGAAACTTTGCTTC-TAMRA | |  |
| *rIl1b* (NM_031512) | |  |  |  |
| Forward | | ACAGAACATAAGCCAACAAGTGGTATT |  |  |
| Reverse | | GTGGGTGTGCCGTCTTTCAT |  |  |
| TaqMan^®^ Probe | | FAM-CAAGGAGAGACAAGCAACGACAAAATCCC-TAMRA |  |  |
| *rNlrp3* (NM_001191642.1) | |  | |  |
| Forward | | GTTTTCCCAGACCCTCATGTTG | |  |
| Reverse | | AGAGACCTCGGCAGAAGCTAGAG | |  |
| TaqMan^®^ Probe | | FAM-CTTCCAGACTGGTGAACTGCTGCCTCA-TAMRA | |  |
| *rNlrp6* (NM_134375.3) | |  |  |  |
| Forward | | CCAAATGCAAGGTGCAGACA |  |  |
| Reverse | | CACAATGACCAGGTAGTGGATCAC |  |  |
| TaqMan^®^ Probe | | FAM-CAGGATACAGATGCCTGGCCTCCAAGA-TAMRA |  |  |
| *rPiezo1* (NM_001077200.2) | |  | |  |
| Forward | | CGTCACCGTCATCATCTCTAAGAAT | |  |
| Reverse | | TGGATGACCCAGCAGAAGTTG | |  |
| TaqMan^®^ Probe | | FAM- TGTTGTCGCTCCTGTCCTGTGTCTTCG-TAMRA | |  |

*CAV1*, caveolin 1; *COL*, collagen; *ELN*, elastin; *IL*, interleukin; *LOX*, lysyl oxidase; *LRRC8A/SWELL1*, leucine rich repeat containing 8 VRAC subunit A, *MMP*, matrix metallopeptidase; *NLRP*, nucleotide-binding oligomerization domain, leucine rich repeat and pyrin; *PIEZO1*, piezo type mechanosensitive ion channel component 1; *SPP1*, osteopontin; *TLR4*, toll-like receptor-4; *TNF*, tumor necrosis factor-α.


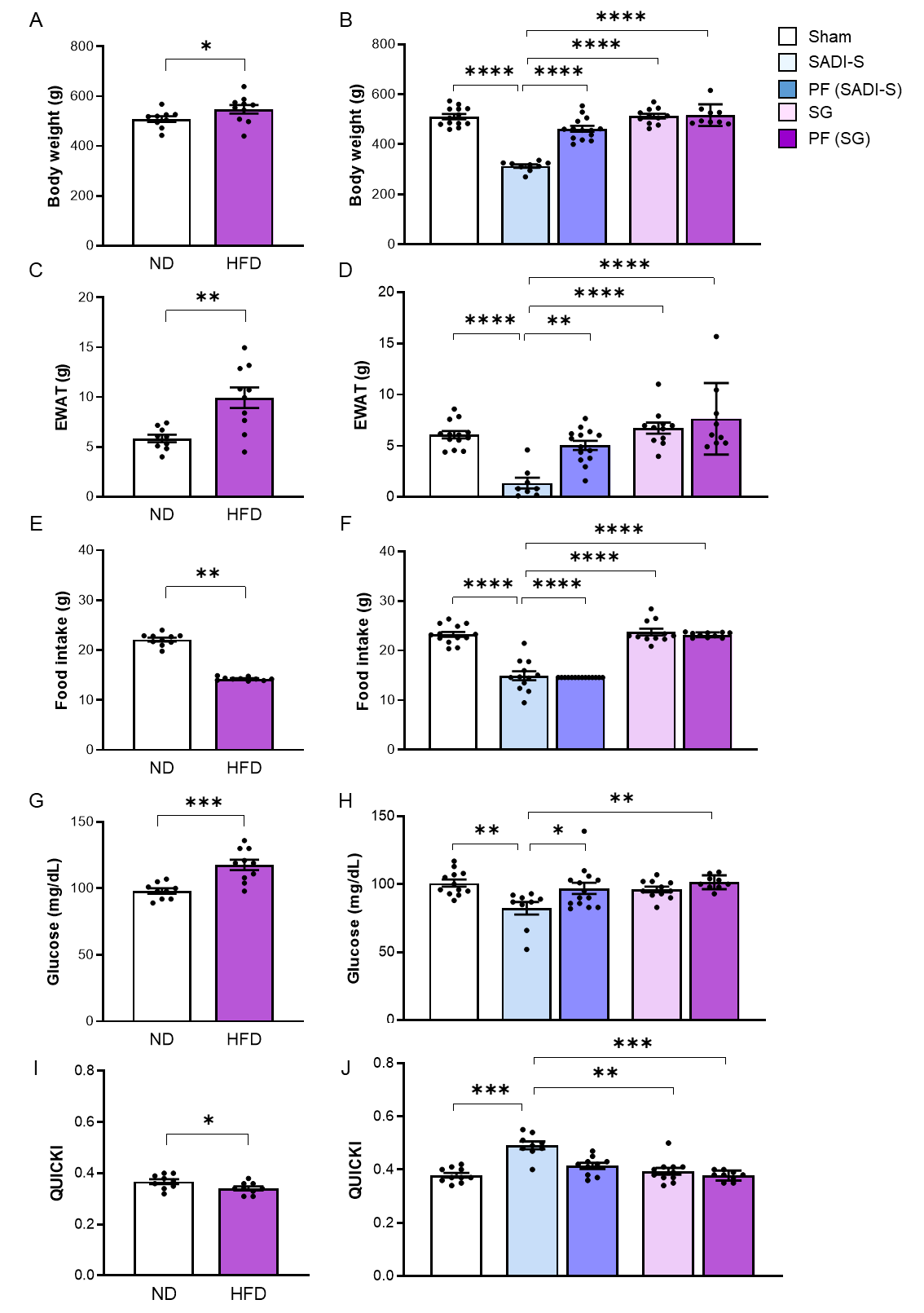


**Supplemental Fig 1.** Body weight, epididymal white adipose tissue (EWAT) weight, food intake, glucose levels and quantitative insulin sensitivity check index (QUICKI) from rats fed a normal diet (ND) or a high-fat diet (HFD) and after being submitted to sham surgery, single anastomosis duodeno-ileal bypass with sleeve gastrectomy (SADI-S), sleeve gastrectomy (SG) and pair-fed (PF). Differences between groups were analyzed by unpaired two-tailed Student’s *t* test as well as by one-way ANOVA followed by Tukey’s tests. ^*^*P*<0.05, ^**^*P*<0.01, ^***^*P*<0.001 and ^****^*P*<0.0001.
